# Fabrication of green colorimetric smart packaging based on basil seed gum/chitosan/red cabbage anthocyanin for real‐time monitoring of fish freshness

**DOI:** 10.1002/fsn3.3574

**Published:** 2023-07-23

**Authors:** Maryam Nadi, Seyed Mohammad Ali Razavi, Dina Shahrampour

**Affiliations:** ^1^ Center of Excellence in Native Natural Hydrocolloids of Iran Ferdowsi University of Mashhad (FUM) Mashhad Iran; ^2^ Department of Food Safety and Quality Control Research Institute of Food Science and Technology (RIFST) Mashhad Iran

**Keywords:** basil seed gum, chitosan, pH indicator, red cabbage, smart packaging

## Abstract

Novel green intelligent films based on basil seed gum (BSG)/chitosan containing red cabbage extract (RCA) (0, 2.5, 5, and 10, % (v/v)) as a colorimetric indicator for food freshness detection were fabricated by casting method. The physicochemical, barrier, mechanical, and antioxidant characteristics, as well as sensitivity to pH and ammonia gas of smart edible packaging films, were investigated. The interaction of anthocyanin extract as a natural dye with biopolymers in films characterized by FTIR spectroscopy and SEM images revealed their suitable compatibility. The film with maximum anthocyanin content (10% (v/v)) appeared robust color changes against various pH and ammonia gas levels. The color of indicator films when exposed to alkaline, neutral and acidic buffers are indicated with green, blue, and red colors, respectively. The DPPH radical scavenging activity of smart BSG/chitosan films improved from 23% to 90.32% with increasing RCA content from 2.5 to 10% (v/v). Generally, the incorporation of RCA in film structure enhanced their solubility, WVP, Δ*E*, turbidity, and flexibility, and reduced tensile strength. The observations successfully confirmed the efficacy of pH‐sensitive indicator smart film based on BSG/chitosan for evaluation of fish spoilage during storage.

## Graphical abstract

Smart films based on basil seed gum/chitosan/red cabbage anthocyanin (RCA) fabricated. RCA concentration significantly affected the antioxidant activity of composite films. Ten‐percent RCA indicated obvious color changes against different pH and ammonia gas levels. Fabricated smart films successfully monitored fish freshness during the storage time.

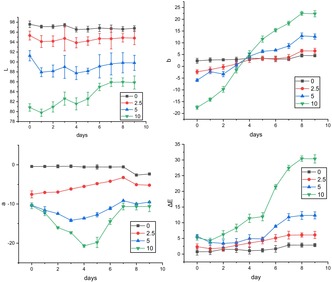

## 1 INTRODUCTION

In the last two decades, intelligent films as a novel food packaging technology have received much research attention. Smart packaging containing indicators can evaluate the internal or external package environment and quickly provide information for the producers or consumers about food quality and safety status without opening the food package. The halochromic indicators respond to changes in temperature, pH, or gases in a package and appear in different colors. In many cases, spoilage of protein‐based foodstuffs along with changes in the pH and production of volatile basic compounds (e.g., dimethyl ammonium, trimethylamine, ammonia, and so on) is due to chemical reactions or microbial growth. Therefore, using colorimetric indicators as a tag or package film with appropriate action speed can be beneficial due to low‐cost, facile manufacturing and easy use for naked‐eye and quick evaluation of food freshness during storage (Li et al., 2021; Yan et al., 2021).

There is an excellent consideration for natural dyes as a practical portion of colorimetric indicators because of their availability, nontoxic, eco‐friendly, renewable, cost‐effective, and safety characteristics instead of artificial colors (Naghdi et al., 2020). Anthocyanins belong to the water‐soluble flavonoids group with phenolic nature, and pH‐sensitive potential, and exhibit various color spectrums because of a change in their structure at various pH levels (Becerril et al., 2021). These flavonoid pigments have revealed the red, blue, and purple colors in many plant sources and have excellent functional activities (Zhao et al., 2023). The anthocyanins are composed of the glycosylated structure of anthocyanidins such as delphinidin, peonidin, cyanidin, malvidin, pelargonidin, and petunidin that are widely found in the environment (Wang et al., 2020). Recently, the addition of anthocyanin extract from various plant sources (red cabbage, red grape, berries, black/purple rice, purple sweet potato, rose, saffron petals, etc.) in polymer‐based films has been used to design pH‐sensing intelligent packaging with color changing properties (Alizadeh Sani et al., 2022; Freitas et al., 2020; Mohseni‐Shahri et al., 2023; Yang et al., 2022; Yong, Liu, et al., 2019; Yong, Wang, et al., 2019; Zhai et al., 2017; Zhang et al., 2021; Zhu et al., 2021). For example, Zhai et al. (2017) used Rosella plant anthocyanins along with starch and polyvinyl alcohol biopolymers to produce calorimetric films and monitor fish freshness. The results revealed significant color changes of films with the increase in the amount of volatile nitrogen in the fish. Also, Kurek et al. (2018) produced chitosan‐based smart films containing blueberry and blackberry pomace extracts as active agents. They reported color changes of films containing blueberry and blackberry from pink to blue–green and red to dark purple during pH changes, respectively. Moreover, the colorimetric indicator film based on butterfly pea (*Clitoria ternatea*) anthocyanin and sago (*Metroxylon sagu*) was developed that revealed acceptable responses to pH changes during chicken breast spoilage by color changes from blue to green (Ahmad et al., 2020). Yan et al. (2021) also incorporated butterfly pea (*Clitoria ternatea*) extract into the chitosan‐based film and observed visible color changes from bluish purple–blue to dark green during fish storage. In another study, Huang et al. (2023) developed a smart film containing purple cauliflower anthocyanin to monitor shrimp freshness during cold storage. Several studies also demonstrated the negative/positive influence of anthocyanins addition on the physical, structural, mechanical, thermal, and functional characteristics of the films (Rodrigues et al., 2021; Yousefi et al., 2019).

Red cabbage is a natural colorant source that is rich in anthocyanins, predominantly mono‐ or di‐acylcyanidin anthocyanins, which have broad color changes in exposure to different pHs (Abedi‐Firoozjah et al., 2022; Musso et al., 2019). Red cabbage anthocyanin received more attention in different studies due to its inexpensive, availability, abundance, and reliable halochromic capacity in comparison with other natural anthocyanin sources (Abedi‐Firoozjah et al., 2022; Liang et al., 2019).

Various synthetic and natural polymers are introduced for the embedding of color agents in food smart packaging. As a unique cationic biopolymer, chitosan achieves by the deacetylation of chitin. Chitosan is a linear polysaccharide composed of D‐glucosamine and N‐acetyl D‐glucosamine units (Dash et al., 2011). Chitosan has been considered an excellent option for the preparation of edible packaging films due to its good biodegradable, antibacterial, and mechanical properties (Yong, Liu, et al., 2019). The presence of amino and hydroxyl groups in its structure can reveal functional properties such as antioxidants and antimicrobials (Duan et al., 2010). The chitosan‐based films have good barrier properties to oxygen and carbon dioxide, but they are permeable to water vapor (Sánchez‐González et al., 2010). The physicochemical and barrier properties of chitosan‐based films can be improved in combination with other hydrocolloids. Several studies have proved the compatibility potential of chitosan in mixing with other biopolymers (Rao et al., 2010; Silva‐Pereira et al., 2015; Zhao et al., 2023).

Basil is a medicinal plant, which is widely cultivated in different regions of the world. Basil seeds contain significant amounts of mucilage, which has favorable functional and physicochemical properties compared to commercial gums to formulate various food products (Razavi & Naji‐Tabasi, 2023; Zameni et al., 2015). Basil seed gum (BSG) macromolecule has two main parts including glucomannan (43%) with a ratio of glucose to mannose of 10:2 and xylan part (24.29%) along with a minor part of glucan (2.31%). The dominant sugar units of BSG consist of glucose, galacturonic acid, rhamnose, mannose, arabinose, glucuronic acid, and galactose (Naji‐Tabasi & Razavi, 2017a, 2017b). BSG exhibited interesting shear thinning behavior and high yield stress, which makes its usage as a thickener and stabilizer in food systems (Hosseini‐Parvar et al., 2010; Rafe et al., 2012). Some of the most important properties of BSG include biodegradability, low production price, hydrophilic and heat‐resistant nature, and favorable rheological properties, which have caused great consideration to edible packaging fabrication (Mohammad Amini et al., 2015; Razavi & Naji‐Tabasi, 2023). Although BSG film has indicated suitable transparency and mechanical properties in previous research, the application of pure BSG film is limited due to high water solubility and weak moisture barrier characteristics. The combination of polysaccharides with other biopolymers as the composite film has been recommended to achieve better bio‐based film with interstitial properties. Since BSG is an anionic glucomannan, it seems to be a suitable option for mixing with cationic biopolymer such as chitosan to produce a composite film.

According to our knowledge, no previous report has been conducted to investigate smart composite chitosan/BSG film characteristics. Therefore, the present study aimed to develop pH‐sensing, eco‐friendly smart composite films based on BSG and chitosan with different contents of red cabbage anthocyanin (RCA) for real‐time evaluation of the freshness of rainbow trout during storage conditions. Besides, the influence of adding RCA on the physicochemical, barrier, mechanical, antioxidant, and antimicrobial characteristics of smart composite films was evaluated.

## 2 MATERIALS AND METHODS

### 2.1 Materials and reagents

The basil seeds and red cabbage were purchased from a local market in Mashhad, Iran. Chitosan was obtained (from shrimp shell, ≥75% (acetylated), *M*_*W*_: 20000 (Da)) from Sigma Aldrich Company, Spain. Acetic acid, hydrochloric acid, sodium chloride, glycerol, ethanol, and 2,2‐diphenyl‐1‐picrylhydrazyl (DPPH) were prepared from Sigma Aldrich Company, Spain.

### 2.2 Extraction of BSG

The optimal condition reported by Razavi et al. (2009) at 68°C, pH 8, and water‐to‐seed ratio of 65:1 with constant stirring for 20 min was applied for BSG extraction. The gum was obtained by passing the swollen seeds through a laboratory extractor (Pars Khazar). A vacuum oven was used for drying extracted gum at 50°C. Finally, it was powdered with a laboratory mill and stored in an appropriate container until the next tests.

### 2.3 Anthocyanin extraction

At first, chopped red cabbage (150 g) was mixed with 80 mL of ethanol–water solution (70:30 ratio) and pH was adjusted to 2. This mixture was kept in a dark place for 24 h at 5°C. After this period, filtration and centrifugation at 6000 rpm for 20 min were done and the supernatant was separated. The red cabbage anthocyanin extract was neutralized using 2.5 M NaOH solution and used for the film preparation (Silva‐Pereira et al., 2015).

### 2.4 Smart composite film fabrication

The chitosan powder (1 g) was dissolved in 100 mL of acetic acid solution (1% v/v) with constant stirring for 2 h at 25°C. Also, BSG powder (0.5 g) was dissolved in distilled water (100 mL) at 50°C. A mixture of both solutions (ratio of 1:1) was prepared, and glycerol (30% w/w of soluble solid weight) as a plasticizer was added. Finally, the different concentrations of RCA (2.5, 5, and 10% (v/v)) were blended with the prepared composite solution. All film‐forming solutions were poured into polyethylene plates (10 cm diameter) and dried at 25°C for 48 h. The composite film without RCA was fabricated as the control sample.

### 2.5 Characterization of the smart composite films

#### 2.5.1 Thickness

The thickness was reported as the average value of 10 random points on smart and control films measured by a digital micrometer (±0.001 mm) (Mitutoyo) (Alizadeh Sani et al., 2022).

#### 2.5.2 Moisture content (MC)

The weight loss percentage of each film piece (2 × 2 cm^2^) after drying (105°C for 24 h) compared to the initial weight was calculated as the moisture content of each film (Shahrampour & Razavi, 2022).

#### 2.5.3 Water solubility (WS)

The solubility of dried films (2 × 2 cm^2^) after immersion in distilled water (50 mL) for 24 h at ambient temperature was determined. At the end of incubation time, the weight of undissolved films after drying at 105°C for 24 h was measured and WS of each film was calculated by the following formula:

(1) $$\mathrm{WS}\;\left(\%\right)=\left(\left(m_{1}–m_{2}\right)/m_{1}\right)\times 100$$

where *m*_1_ is the initial dried film mass (g) and *m*_2_ is the dried undissolved film mass (g) (Shahrampour & Razavi, 2022).

#### 2.5.4 Water vapor permeability (WVP)

The water vapor permeability (WVP) of films was determined as described method by Moghadam et al. (2021). Briefly, the film pieces were placed on glass cell openings containing dried calcium chloride (0% RH) and sealed tightly and moved to the desiccator containing NaCl saturation solution (75% RH). The mass changes of each cell were recorded for 4 days and WVP was estimated by using Equation (2):

(2) $$\mathrm{WVP}=\left(\text{∆W}.x\right)/\left(\text{∆t}.A.\text{∆P}\right)$$

where (*∆W*/*∆t*) is the mass change rate (g/s), *x* is the film thickness (m), *A* is the film area (m^2^), and *∆P* is the partial pressure difference between two surfaces of each film (Pa).

#### 2.5.5 Color and opacity characterization

The photographic images of films were recorded by a digital camera (Canon, 16.5 MP), and color indexes of the film samples such as *L* (lightness), *a* (red‐green), and *b* (yellow–blue) were determined by Image J. software (1.52v, USA). Also, the total color difference (Δ*E*) index was calculated using Equation (3):

(3) $$\Delta E=\surd \left({\left(L\times -L\right)}^{2}+{\left(a\times -a\right)}^{2}+{\left(b\times -b\right)}^{2}\right)$$

Each film was cut with specific dimensions (0.7 × 1.5 cm^2^) and placed in a plastic cell and its absorption at 600 nm was recorded by Ultraviolet–Visible spectrophotometer (Jenway Ltd). The following equation was used for the measurement of film opacity (Alizadeh Sani et al., 2022):

(4) Opacity=A/T

where *A* is the adsorption at 600 nm and *T* is the film thickness (mm).

#### 2.5.6 ATR‐FTIR spectroscopy

Attenuated total reflectance–Fourier transforms infrared (ATR‐FTIR) spectroscopy (Nicolet 370, Thermo Nicolet) was used to reveal the structural interactions of BSG/chitosan and red cabbage anthocyanin within the films. The spectra were recorded in the range of 4000–400 cm^−1^ at 25°C temperature. Data were analyzed and graphs were drawn using the Orginlab software, 2019.

#### 2.5.7 Mechanical properties

The conditioned films (at 25°C and 53% RH for 48 h) were cut with specific dimensions (10 × 1.5 cm^2^), and fixed between two grips of a texture analyzer (Brookfield Engineering). The initial distance of grips was set to 50 mm and a strain rate of 50 mm/min was operated. The mechanical parameters of films such as tensile strength (TS), percentage of elongation at the breakpoint (EB %), and elastic modulus (E) were calculated according to the ASTM D882‐02 standard (ASTM, 2002) through the following formulas.

(5) $$\mathrm{TS}=F_{\max}/A$$

(6) $$\mathrm{EB}\%=\left(\Delta L/L_{0}\right)\times 100$$

(7) $$E=\sigma /\varepsilon =\left(F/A\right)/\left(\Delta L/L_{0}\right)={\mathrm{FL}}_{0}/A\;\Delta L$$

where *F*_max_ = the force at the breakpoint (N), *A* = the film area (mm^2^), Δ*L* = *L* − *L*_*0*_, *L* = the film length at the breakpoint (mm), *L*_*0*_ = the initial film length (mm), *σ* = stress (N/mm^2^), and *ε* = strain (−).

#### 2.5.8 Microstructure analysis

The microstructure analysis of films was carried out by a scanning electron microscope (MIRA 3 TE SCAN) that operated at 6 kV under vacuum conditions after coating a small piece of each film with gold for more conductivity.

##### Determination of sensibility to different pH buffers

The efficacy of fabricated smart films as a pH indicator was evaluated. Briefly, the anthocyanin‐loaded films were immersed in buffer solutions with different pH (2, 7, and 12), and the photographic images of the films' color changes after 5 min were recorded by a digital camera (Canon, 16.5 MP) (Moghadam et al., 2021).

#### 2.5.9 Evaluation of sensibility to volatile ammonia gas

The colorimetric response of films containing various anthocyanin concentrations was examined after incubation of film pieces above a glass container containing ammonia solution (8 mM) for 20 min at room temperature. The photographic images of films' color changes were recorded by a digital camera (Canon, 16.5 MP).

### 2.6 Measurement of functional activity of smart films

#### 2.6.1 Antioxidant activity

The DPPH free radical scavenging activity of smart films was calculated as antioxidant potential based on the method described by Moghadam et al. (2021).

#### 2.6.2 Antimicrobial activity

The antimicrobial potential of smart films against *Staphylococcus aureus* (PTCC 1431) and *Escherichia coli* (PTCC 1399) as gram‐positive and gram‐negative pathogenic bacteria, respectively, was determined by the disk diffusion method. At first, 100 μL of a fresh overnight culture of bacteria (10^8^ CFU/mL) was poured on the nutrient agar surface and a disk of each film sample was transferred to it. After spending 24 h at 37°C, the diameter of the inhibition zone around the films was measured by a ruler and reported in mm (Alizadeh Sani et al., 2022).

### 2.7 Monitoring of fish freshness

The fresh rainbow fish was bought from a local market (Mashhad, Iran) and immediately transferred to the laboratory. The fish pieces (20 g) were placed in sterile Petri dishes and each smart film (2 × 2 cm^2^) was fixed on the internal space of its lids. These plates were incubated at 25 and 4°C for 1 and 9 days, respectively. The photographic images of films' color changes were recorded by a digital camera (Canon, 16.5 MP), and color indexes were determined by the Image J. software (1.52v, USA). Moreover, the changes in pH, microbial loaded, and TVN‐B of fish samples were determined during analysis. Briefly, the fish sample was homogenized with 10 volumes of deionized water, and pH was measured using a pH meter (Metrohm, Swiss). For evaluation of the total viable microbial count (TVC) of the samples, 10 g of fish was transferred to a sterile ringer solution (90 mL) and well homogenized. Then, serial dilutions were prepared and used for pour‐plate culture on plate count agar (PCA). The number of colonies on the medium after 48 h of incubation at 37°C was reported as TVC.

Moreover, total volatile nitrogen base (TVN‐B) was evaluated by AOAC (1990) method. Briefly, a fish sample (10 g) was added to 300 mL of distilled water containing 2 g of magnesium oxide in a heating flask. The receiving flasks contained 25 mL of boric acid (2%) and a few drops of methyl red. Afterward, the flasks used for the heating and receiving were connected to an evaporator until distillation was stopped. Finally, by using sulfuric acid (0.1 N), the contents of the receiving flask were titrated to the endpoint, and the TVB‐N was determined as follows (AOAC, 1990; Yehia et al., 2021):

$$\mathrm{TVN}-B=\left(V\times N\times 100\times 14\right)/W$$

where V is the volume of sulfuric acid (mL), N is the normality of sulfuric acid, and W is the weight of the sample in grams.

### 2.8 Statistical analysis

Statistical analysis of data was done using SPSS software (Version 16, SPSS Inc). For this purpose, a completely random design and one‐way analysis of variance (ANOVA) were used. To determine the significant difference between the averages, Duncan's test method was applied at the statistical level of *p* < .05.

## 3 RESULTS AND DISCUSSION

### 3.1 Thickness

Film thickness can directly affect several mechanical characteristics, permeability, and transparency of films (Yong, Liu, et al., 2019). According to Table 1, the thickness of the films increased by enhancing RCA content, which was significant only at 10% RCA (*p* < .05). The tested film thickness ranged from 0.056 mm (control film) to 0.079 mm (film containing 10% (v/v) RCA). This effect could be related to the higher amount of solid matter in the film. These findings were consistent with Park et al. (2022) research. Yong, Wang, et al. (2019) reported enhancing film thickness after the incorporation of 5% black and purple rice extract into chitosan‐based films. No changes in the thickness of chitosan films containing different amounts of RCA were obtained in the research of Chen et al. (2021). This observation is probably related to the low content of cabbage extract in chitosan‐based films.

**TABLE 1:** The physicochemical and barrier properties of smart films containing different concentrations of RCA (RCA).

RCA content (%)	Thickness (mm)	Moisture content (%)	Water solubility (%)	WVP (×10^−10^ g Pa^−1^m^−1^s^−1^)
0	0.056 ± 0.015^a^	33.01 ± 1.50^a^	26.34 ± 1.66^c^	2.763 ± 0.119^b^
2.5	0.058 ± 0.005^a^	32.45 ± 0.23^ab^	30.55 ± 0.13^b^	3.034 ± 0.021^b^
5	0.063 ± 0.001^a^	30.56 ± 4.84^ab^	31.71 ± 1.65^b^	3.442 ± 0.424^ab^
10	0.079 ± 0.005^b^	29.20 ± 0.63^b^	35.26 ± 0.39^a^	4.026 ± 1.156^a^

*Note*: Data are means ± SD (*n* = 3). Different letters in each column indicate a significant difference (*p* < 0.05) according to Duncan's means post hoc comparison.

### 3.2 Moisture content (MC)

Moisture content is a significant indicator to determine the film's physical integrity when exposed to conditions with high humidity (Peralta et al., 2019). Table 1 shows that with increasing RCA from 2.5% to 10% (v/v), the MC of films decreased from 32.45% to 29.20%, respectively. There was only a significant difference between the control film and the smart film containing the highest content of extract (*p* < .05). Anthocyanins contain many hydroxyl groups that establish hydrogen bonds with hydrophilic groups of film compounds, which limits their interaction with water molecules (Yong, Liu, et al., 2019). Therefore, increasing an anthocyanin extract content could decrease the MC of a film. A similar report was presented by Wang et al. (2019). They found that the MC of the chitosan film containing black soybean husk extract gradually reduced from 31.01% in the control film to 21.29% in the film containing 15% extract. In similar, Yong, Wang, et al. (2019) stated that more moisture content in chitosan film containing 5% of purple‐fleshed sweet potato extract compared to its 15%. According to Khazaei et al. (2014) report, the BSG film contained lower moisture content in contrast to the control chitosan/BSG composite film in the recent study (Khazaei et al., 2014).

### 3.3 Solubility in water

Solubility in water indicates the film's moisture sensitivity. A smart film with high water sensitivity quickly dissolves in water solutions, which leads to the loss and release of its colorimetric agents (Ozdemir & Floros, 2008). Table 1 shows that the addition of RCA in all levels (2.5, 5, and 10% (v/v)) could significantly enhance the solubility of the films (*p* < .05). The solubility of the studied films ranged from 26.34% to 35.28%. Prietto et al. (2017) reported an increase in the solubility of corn starch films from 21.6% to 48.6% after the incorporation of red cabbage anthocyanin due to the increase of hydrophilic points in the film structure. Similar findings were presented by Yong, Wang, et al. (2019) regarding an increase in solubility from 17.04% to 26.47% in chitosan film without extract and chitosan film containing 10% purple sweet potato extract, respectively. In contrast, Wu et al. (2020) reported lower solubility for an active smart film based on konjac glucomannan/oxidized chitin nanocrystals and RCA in comparison with the control film. In addition, blending chitosan with BSG solution could decrease the solubility of the film. As a result, the control composite film based on chitosan/BSG indicated lower solubility in water (26.34%) compared to pure basil film reported in previous studies (51.93%) (Khazaei et al., 2014).

### 3.4 Water vapor permeability (WVP)

The permeability property of packaging films is an essential factor to maintain the quality and security of foodstuffs. According to Table 1, the WVP of films increased with RCA addition to the film solution. The smart film containing 10% (v/v) RCA indicated a maximum WVP value. However, no significant difference between control and smart films at levels of 2.5 and 5 (v/v, %) of the extract was observed (*p* > .05). This occurrence can be related to the hydrophilic character of anthocyanin with several hydroxyl groups and enhancing hydrophilic points in the composite film (Wu et al., 2020). In general, the WVP of packaging films can be influenced by various factors such as the uniformity of the polymer chain, and the hydrophilicity or hydrophobicity of other additives (Wardana & Widyaningsih, 2017). The results of Liang et al. (2019) study revealed a significant increase in the WVP of a film based on *Artemisia* gum after embedding 15% RCA in the film structure. In contrast, Chen et al. (2020) reported no significant influence after the addition of natural dyes on the WVP of composite starch/polyvinyl alcohol films. The decline in the binding affinity toward water molecules and improvement of barrier properties was observed in composite chitosan/oxidized‐chitin nanocrystal films containing different amounts of red cabbage anthocyanin compared to the control film (Chen et al., 2021). Moreover, a slight increase was observed in the WVP of the control composite film in comparison with each pure biopolymer in the previous research works (Khazaei et al., 2014; Soares et al., 2022). It was probably due to the new interaction between the two biopolymers and the creation of empty spaces.

### 3.5 Color and opacity properties

The color indexes of films including L (lightness), a (green/red), b (blue/yellow), the total color difference (Δ*E*), and their images are illustrated in Table 2. Generally, the increase of RCA content in film structure led to a significant reduction of *L*, *a*, and *b* indexes and color changes of films from light blue to dark blue color (*p* < .05). The total color difference (Δ*E*) indicates the color changes of anthocyanin‐loaded films compared to the control film which was significantly increased from 10.2 to 28.51 in smart films containing 2.5% and 10% of anthocyanin content, respectively (*p* < .05). The opacity was also enhanced after entrapping of RCA in the film matrix. The lowest (2.44) and highest (4.24) opacity belonged to the control film and smart film containing 10% RCA extract, respectively. The opacity is known as a positive aspect of packaging film to limit ultraviolet light transmission and protect food from adverse changes and lipid oxidation (Jiang et al., 2020). Cheng et al. (2022) observed an increase in starch film opacity from 0.39 to 1.34 after the addition of 40% RCA. Kurek et al. (2018) found that Δ*E* and opacity of chitosan film variably increased after the addition of blueberry and blackberry anthocyanin extracts. Therefore, the color property of anthocyanin‐loaded films is closely dependent on the composition and content of anthocyanin. Moreover, the results of this research revealed an improvement in the lightness of BSG film (76.57) (Khazaei et al., 2014) after blending with chitosan in the composite film (97.88).

**TABLE 2:** Color indexes and optical properties of smart films containing different concentrations of red cabbage extract (RCA).

RCA content (%)	L	a	b	Δ*Ε*	Opacity	Image of films
0	97.88 ± 0.22^a^	‐0.48 ± 0.73^a^	1.97 ± 0.61^a^	0^d^	2.44 ± 0.80^b^	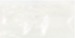
2.5	94.93 ± 0.39^b^	−9.18 ± 0.85^b^	−2.43 ± 0.01^b^	10.20 ± 0.99^c^	3.41 ± 0.43^ab^	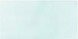
5	92.04 ± 0.42^c^	−10.90 ± 0.62^b^	−5.40 ± 0.29^c^	14.05 ± 0.31^b^	3.62 ± 1.62 ^ab^	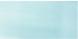
10	80.18 ± 0.73^d^	−10.55 ± 3.24^b^	−17.89 ± 0.41^d^	28.51 ± 2.09^a^	4.24 ± 1.01^a^	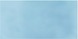

*Note*: Data are means ± SD (*n* = 3). Different letters in each column indicate a significant difference (*p* < .05) according to Duncan's means post hoc comparison.

### 3.6 Mechanical properties

**FIGURE 1:**
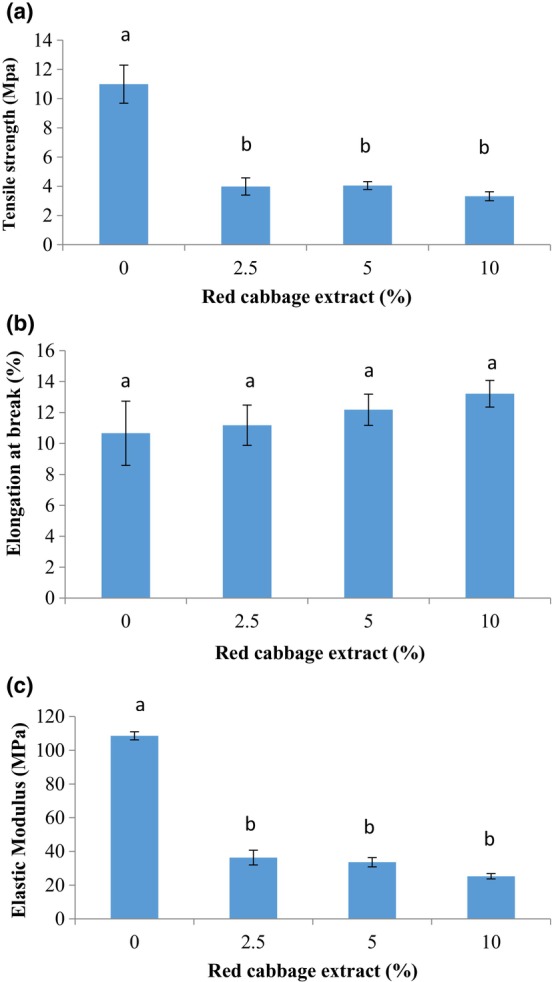
The mechanical characteristics of films based on BSG/chitosan containing different concentrations of RCA (0, 2.5, 5, and 10 (%v/v)); (a) tensile strength, (b) elongation at break (%), and (c) elastic modulus.

The results of the tensile strength (TS), elongation at break point (%) (EB %), and elastic modulus (EM) are depicted in Figure 1. The tensile strength of composite chitosan/BSG films significantly diminished with the RCA addition (*p* < .05). There was no significant difference between smart films containing different levels of RCA (*p* > .05). The highest and lowest tensile strength values were obtained for the control film (10.99 MPa) and 10% red cabbage‐loaded film (3.32 MPa), respectively. Moreover, the elastic modulus of chitosan/BSG film significantly decreased from 108.57 MPa to 25.26 MPa after the addition of RCA. On the contrary, a slight enhancement of elongation at break (%) was observed after RCA incorporation in composite films. However, these changes were not significant (*p* > .05). The obtained results are probably due to disturbing the compact network of the film and create weaker interactions with changes in hydrogen bonds in the polymer chain (Liang et al., 2019). In accordance with our findings, Pereira Jr et al. (2015) reported a reduction in the tensile strength of indicator film based on chitosan/polyvinyl alcohol/red cabbage anthocyanin while the elongation of the films was not affected by the presence of anthocyanin. Liang et al. (2019) observed a decrease in the tensile strength and an increase in elongation (%) for carboxymethyl cellulose/Artemisia gum composite film after the addition of 15% (w/v) RCA. The presence of RCA (1.2% w/v) in chitosan/oxidized‐chitin nanocrystal composite films could improve the mechanical resistance and flexibility of the films (Chen et al., 2021). In addition, the tensile strength (25.22 MPa) and elongation (%) (32.5%) of BSG film in Khazaei et al. (2014) study was more than our composite film based on chitosan/BSG. This difference is probably related to the creation of weaker interactions between two biopolymers' chains in the composite film structure.

### 3.7 Functional chemical groups

**FIGURE 2:**
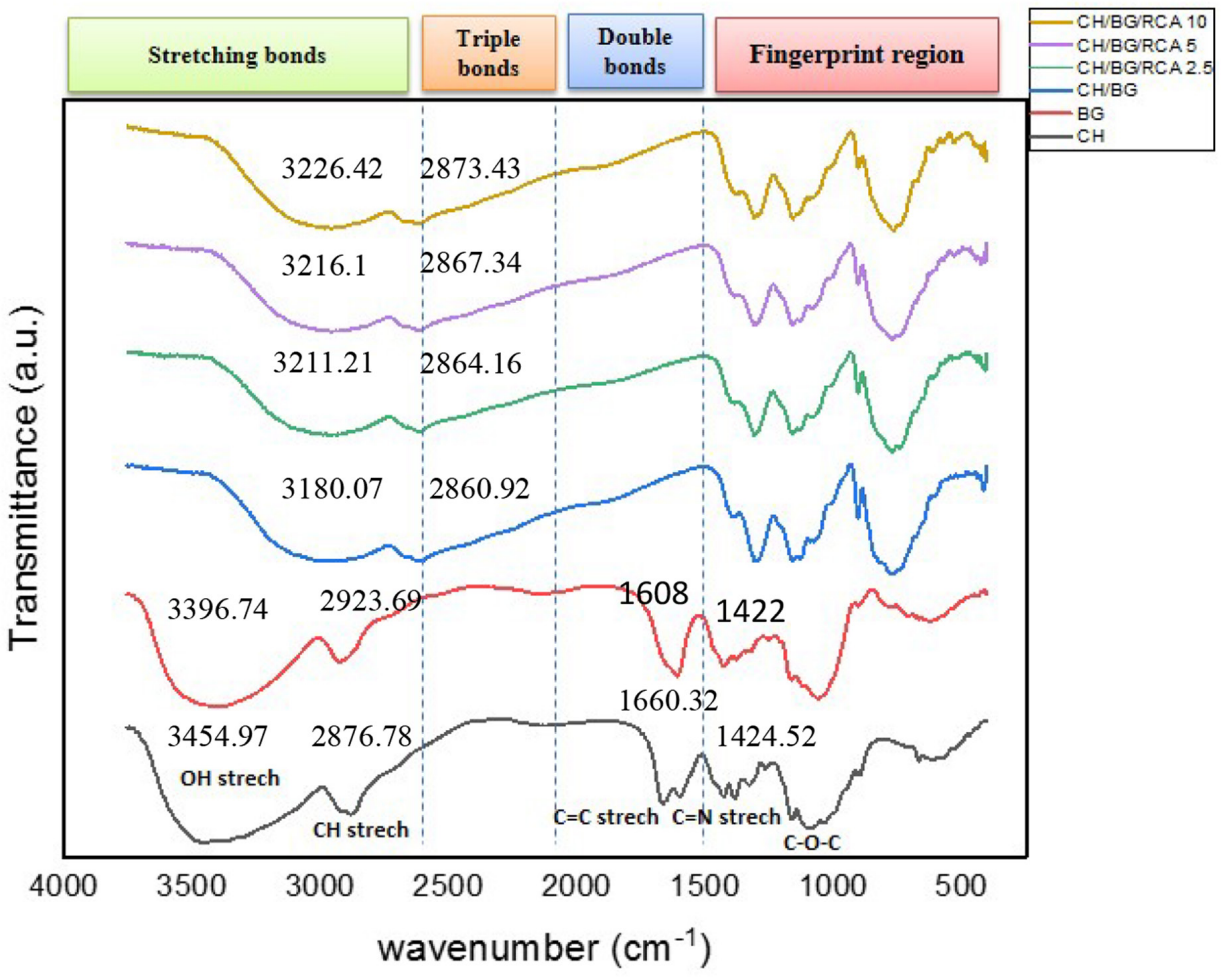
FTIR spectra of BSG/chitosan composite films containing different concentrations of RCA (0, 2.5, 5, and 10 (%v/v)).

The intermolecular interactions between biopolymers and RCA in the films' structure were analyzed by the ATR‐FTIR spectrum. The spectrum is distributed in four general regions, including stretching bonds (2500–4000 cm^−1^), triple bonds (2000–2500 cm^−1^), double bonds (1500–2000 cm^−1^), and fingerprint region (600–1200 cm^−1^) which each material in this region has a unique absorption peak (Guo et al., 2011). Figure 2 demonstrates the ATR‐FTIR spectra of the chitosan, BSG, and their composite film and RCA‐loaded smart films.

The spectra of chitosan indicated the bonds of 3454 cm^−1^ (O‐H stretching), 2876 cm^−1^ (bending vibration of C‐H), 1660 cm^−1^ (amide I and C=O stretching), 1424–1324 cm^−1^ (CH_2_ bending vibration), and 710 cm^−1^ (CH bending vibration). On the other hand, the spectra of BSG exhibited the main bonds 990–1150 cm^−1^ (C‐O, C‐O‐C stretching), 1400 and 1600 cm^−1^ are assigned to C‐O‐O symmetric and asymmetric and related to the presence of uronic acid and 2800–3000 cm^−1^ (bending vibration of C‐H) (Naji‐Tabasi & Razavi, 2017a, 2017b). After the preparation of composite film based on BSG/chitosan, a difference was presented in the bonds' positions. All bonds shifted to the right side of the graph and OH stretching vibrations bond became wider and the intensity of bonds around 1608 and 1422 increased. Also, the observation indicated that no significant interaction was formed between BSG/chitosan film and red cabbage anthocyanin. Only some differences in bonds' positions and peak intensity were revealed in the composite chitosan/BSG films in the presence of RCA. The wide peak located at 3180.07 cm^−1^ opened slightly and shifted to 3226.42–3211.21 cm^−1^, which is related to OH stretching vibrations bond involved in the hydrogen bond. The stretching and bending vibrations of the CH‐ and CH_2_ groups that are present in anthocyanin aromatic rings were revealed by absorption peaks in all smart films in the range of 2800–3000 cm^−1^ (Kang et al., 2011). C‐H stretching bond in the control film slightly shifted from 2860.92 cm^−1^ to 2864.16–2873.43 cm^−1^ in anthocyanin‐loaded films. These absorption peaks revealed the formation of new hydrogen bonds between anthocyanin and chitosan/BSG, indicating the integration of the RCA in the polymers' matrix (Freitas et al., 2020). Similar observations were also reported by Chen et al. (2021) about a minor shift of the absorption peak at 3388 cm^−1^ after the incorporation of RCA in composite chitosan/oxidized chitin nanocrystal which was assigned to the O‐H stretching vibration and confirmed the formation of hydrogen bonds between the film compounds and red cabbage anthocyanin.

### 3.8 Microstructure

**FIGURE 3:**
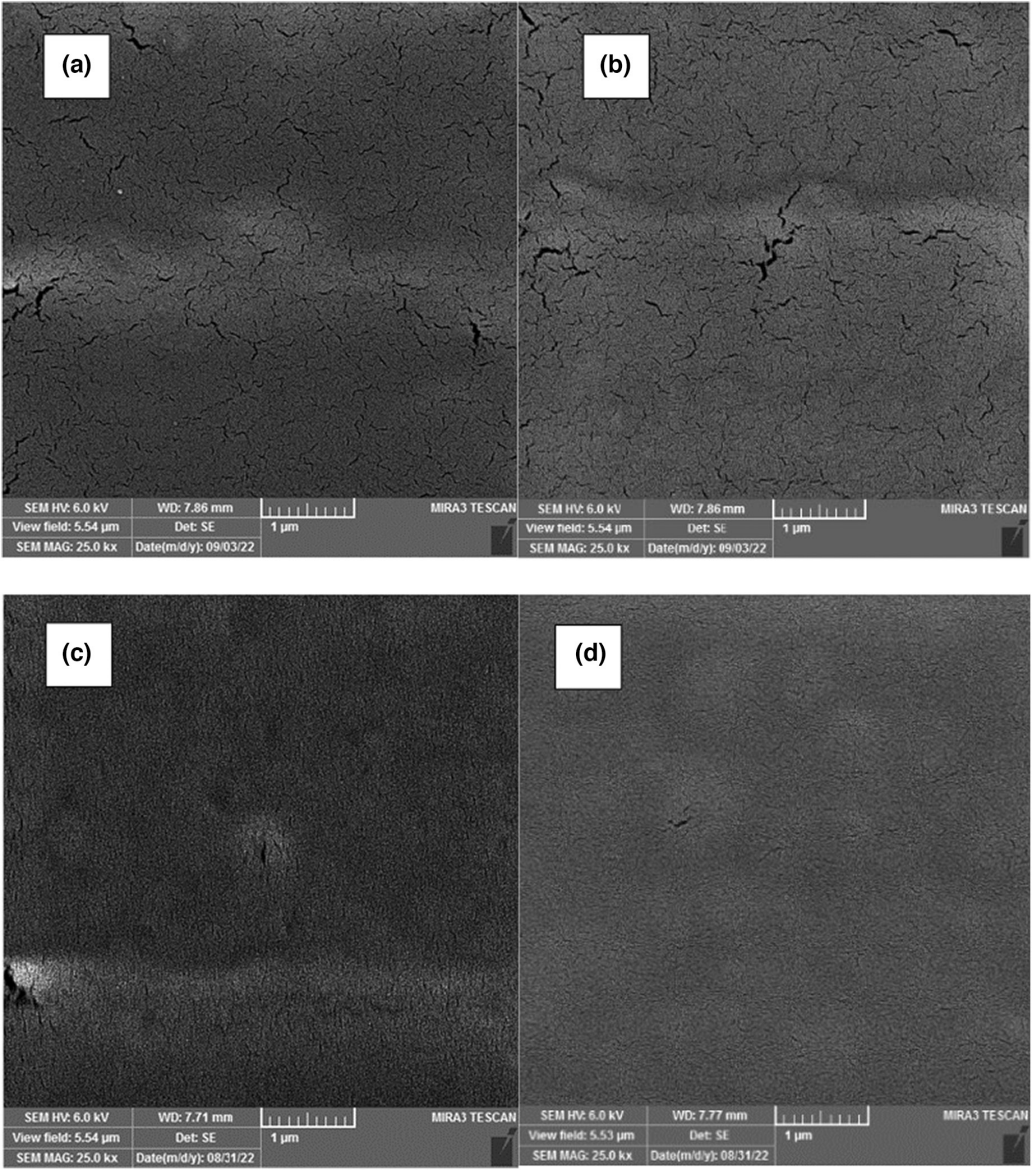
The microstructure images of BSG/chitosan composite films containing different concentrations of RCA: (a) 0, (b) 2.5, (c) 5, and (d) 10 (%v/v).

The SEM images related to the surface of composite films based on chitosan/BSG containing different concentrations of RCA (0, 2.5, 5, and 10, v/v %) are illustrated in Figure 3. A nonuniform structure, and many cracks were observed in the control film without RCA content. The structural character of the film improved in the presence of RCA. The composite film with maximum content of RCA (10%, v/v) revealed a dense and relatively uniform structure with no gaps due to plasticizing character of anthocyanin. These findings confirmed the compatibility between the polymers and anthocyanin that well dissolved in the film matrix. Similar observations after the addition of sweet potato and red cabbage anthocyanin extracts in starch/carboxymethylcellulose and chitosan/oxidized chitin nanocrystal films, respectively, are reported in previous studies (Chen et al., 2021; Jiang et al., 2020).

### 3.9 Antioxidant activity

**FIGURE 4:**
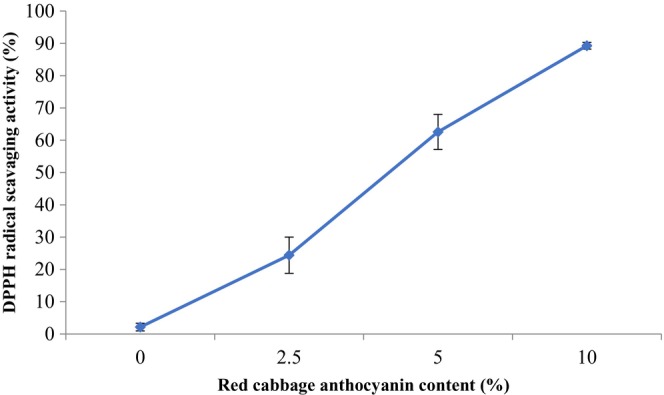
The antioxidant activity of BSG/chitosan composite films containing different concentrations of RCA (0, 2.5, 5, and 10 (%v/v)).

Figure 4 depicts the DPPH radical scavenging activity of composite films with various RCA contents. The results confirmed significant development of the DPPH radical scavenging activity of the films (*p* < .05) with increasing RCA content in the film structure. The lowest and highest antioxidant activity (%) values belonged to the control film (2.125%) and smart film with 10% RCA content (89.28%), respectively. In this regard, the addition of 40% red cabbage anthocyanin in starch film‐forming solution enhanced the antioxidant potential of the film from 2.92% to 45.21% [40]. Also, the enrichment of mung bean protein film with 10% *Echium amoenum* anthocyanin improved its antioxidant activity from 4.66% to 55.62% (Moghadam et al., 2021). These findings can be related to the strong antioxidant nature of natural anthocyanins (Wu et al., 2020). This ability can be attributed to polyphenols' nature, which suppresses free radicals by donating hydrogen atoms (Yong, Liu, et al., 2019).

### 3.10 Antimicrobial activity

The antimicrobial potential of smart films with various RCA contents against *S. aureus* (G+) and *E. coli* (G‐) bacteria was investigated in the diffusion method. No inhibition halo was observed around film pieces that revealed no antimicrobial activity of smart films against the tested bacteria. Probably, the low concentration of anthocyanin or its interactions with the film components can be the reason for its limited release and lack of antimicrobial activity in the films (Musso et al., 2019). Similarly, Musso et al. (2019) stated no antibacterial and antifungal activity of gelatin‐based film containing RCA.

### 3.11 pH sensitivity of films

**FIGURE 5:**
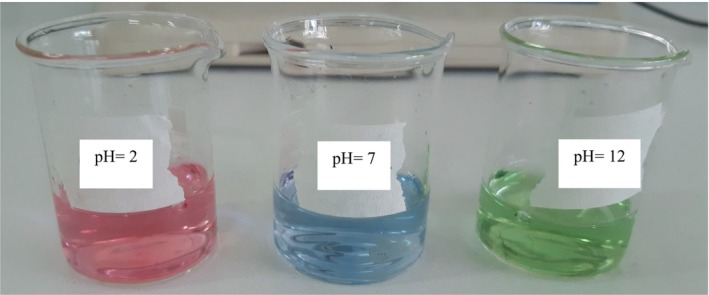
Color changes of red cabbage anthocyanin extract in acidic, neutral, and alkaline buffer solutions with different pH levels.

**FIGURE 6:**
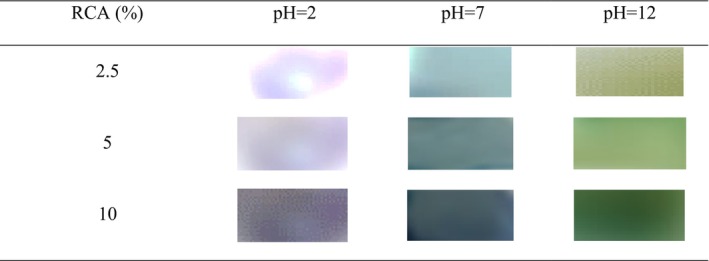
The pH sensitivity of smart films based on BSG/chitosan containing different concentrations of RCA (0, 2.5, 5, and 10 (%v/v)).

The color changes of RCA and pH‐sensitive films in buffer solutions with various pH levels (2, 7, and 12) are shown in Figures 5 and 6, respectively. The color of the anthocyanin solution changed from dark pink at pH 2 to green at pH 12. Also, the color of the films changed from purple‐pink color at pH 2 to green color at pH 12, which the changes were easily detected by the naked eye. It should be mentioned that with the increase of anthocyanin content, the color intensity in the films increased. The color variation of smart films is attributed to the presence of anthocyanin and their structure change during pH changes. In this regard, the color changes of anthocyanin extract of *Echium amoenum* flower from red in an acidic environment to dark green in an alkaline environment were obtained in Moghadam et al. (2021) research. Following our observations, the color changes of smart films based on konjac glucomannan/oxidized chitin nanocrystals or chitosan/PVA containing red cabbage anthocyanin were pink‐red to green after exposing to acidic and alkaline pH, respectively (Pereira Jr et al., 2015; Wu et al., 2020). These results distinguish the important role of active and sensitive components such as anthocyanin in various polymeric films to distinguish the environmental changes of food packaging.

### 3.12 Ammonia gas sensitivity of films

**FIGURE 7:**
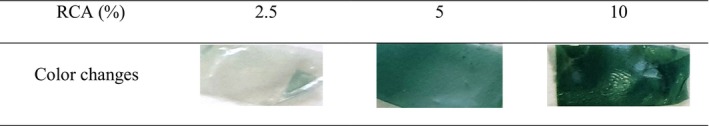
Color changes of smart BSG/chitosan composite films containing different concentrations of RCA (0, 2.5, 5, and 10 (%v/v)) after 20‐min exposure to ammonia gas.

The stability of anthocyanins is affected by pH value; light, chemical components, storage temperature, and oxygen concentration, and their structural variation can be distinguished by color changes (Liu et al., 2021). In this study, the simulated condition was designed to evaluate the sensitivity of colorimetric film to volatile nitrogen compounds during fish meat spoilage. Figure 7 demonstrates the reaction of colorimetric films in the presence of ammonia gas. The color of anthocyanin‐loaded chitosan/BSG films after exposure to ammonia gas for 20 min changed to green. It should be noted that the created green color intensity in the film containing 10% RCA was higher than in films with lower loaded anthocyanin. The mechanism of the color change of smart films is related to a combination of volatile NH_3_ with H_2_O content of films and the final formation of NH_4_^+^ and OH^−^ that causes an alkaline environment and changes anthocyanin structure and its exhibited color (Chen et al., 2020; Zhai et al., 2017). Similarly, Zhai et al. (2017) reported the color changes of sour tea anthocyanin‐loaded starch/polyvinyl alcohol film exposed to ammonia from red to green. Chen et al. (2021) observed the color change of chitosan/oxidized chitin nanocrystals containing red cabbage anthocyanin films exposed to ammonia from red to green/earthy yellow as a result of the change in the structure of anthocyanin immobilized in the film. Liu et al. (2021) also recorded light green color for polyvinyl alcohol/sodium carboxymethyl cellulose colorimetric film containing red cabbage anthocyanin exposed to ammonia. Conclusively, the results of this test confirmed that the RCA‐loaded chitosan/BSG films are sensitive to ammonia volatile vapor, and their response as film color changes occurred in a short time. Therefore, these smart films are suitable for detecting the volatile nitrogen compounds produced in seafood packages.

### 3.13 Fish freshness evaluation by smart films

**FIGURE 8:**
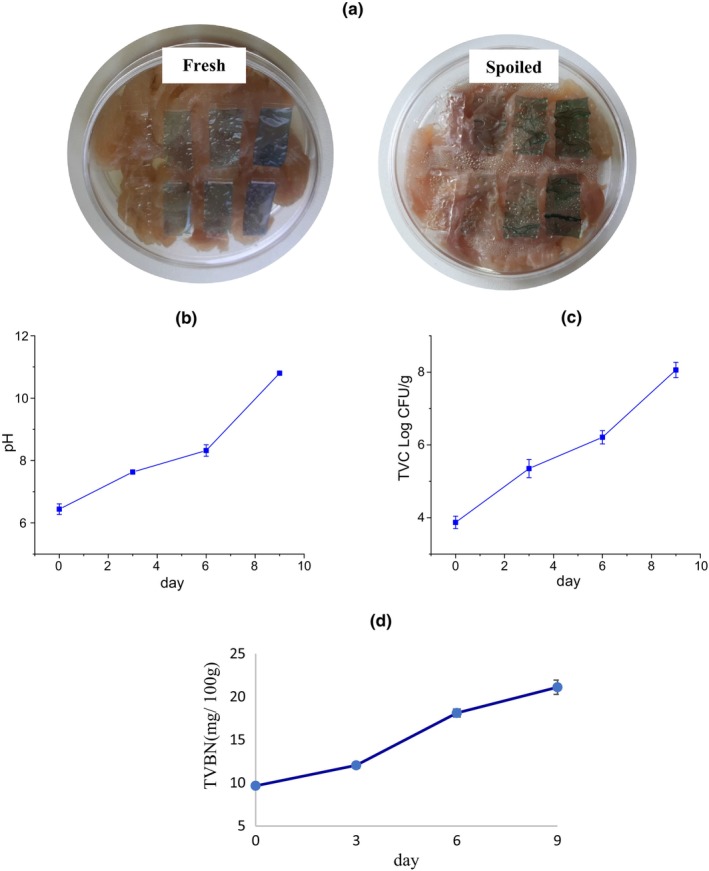
Application of smart films to monitoring fish freshness: (a) appearance of films in the presence of fresh and spoilage packaged fish, (b) Total microbial count, (c) pH values, and (d) TVB‐N of fish during the storage period.

The total viable count (TVC) of microorganisms loaded in fish fillet samples at the initial and end of storage time and different temperatures were determined to evaluate the fish spoilage. The count of 7 log CFU/g was known as a critical microbial load for the start of microbial spoilage of food (Senter et al., 2000). The spoilage microorganisms grow faster as the storage time increases and create different metabolites, which are responsible for the bad smell, taste, and change in color and texture of foodstuff (Huang et al., 2019). In the recent study, the initial TVC on fresh fish was counted at 3.875 Log CFU/g which reached 8.385 Log CFU/g after 24 h of storage at 25°C, while the final population of loaded microorganisms in fish samples after 9 days of storage at a refrigerator (4°C) was estimated as 8.06 Log CFU/g (Figure 8). According to these observations, the initial microbial population on the fish sample surface grew so faster at ambient temperature in comparison with low temperatures. The microbial spoilage was relatively postponed by storage at refrigerator temperature. These final microbial counts denoted fish spoilage after the suggested storage time in the present study. Huang et al. (2019) observed an increase in TVC of fresh fish samples from 4.61 Log CFU/g to 7.23 Log CFU/g and 7.11 Log CFU/g after 12‐h storage at 25°C and 5 days of storage at 4°C, respectively. Similarly, Jiang et al. (2020) reported the correlation between color changes of indicator films of carboxymethyl‐cellulose/starch/purple sweet potato anthocyanin and microbial spoilage of fish during cold storage.

The TVB‐N is a chemical quality assessment parameter for the determination of ammonia, dimethyl‐ and trimethylamine in fish, which is related to the activity of spoilage bacteria and internal enzymes. The TVB‐N concentration in freshly caught fish is usually 5–20 mg/100 g (Rezaei & Hosseini, 2008). A level of 35 mg TVB‐N/100 g of fish muscle is usually regarded as the limit of acceptability, beyond which the fish can be considered as spoiled. In the present research, the initial TVB‐N content of fish was 9.66 mg/100 g and changed to 22.96 and 21.11 mg/100 g after 24‐h and 9‐day storage at ambient (25°C) and refrigerator temperature (4°C), respectively (Figure 8d). These observed changes were consistent with the increase in microbial load and pH of the fish, which was confirmed by the color changes of smart film exposed to the fish.

Huang et al. (2019) also reported the enhancement of TVB‐N from 6.3 mg/100 g to 22.4 mg/100 g and 20.1 mg/100 g, after storage of fish at different conditions such as 25°C for 20 h and 4°C for 6 days, respectively. Similarly, Chen et al. (2020) observed increase of TVB‐N content of carp samples from 7.44 mg/100 g to >20 mg/100 g after 8 days of storage at 4°C. Chen et al. (2021) reported 21.10 mg/100 g for TVB‐N value after 36 h of fish storage at 25°C, which indicated that the sample was partially spoiled and it reached 35.09 mg/100 g, which was no longer suitable for consumption. The changes of TVB‐N from 6.56 to 36.79 mg/100 g, indicating the complete corruption of the fish at ambient temperature followed by color changes of intelligent film based on carboxymethyl‐cellulose/starch and purple sweet potato, have been reported in previous research (Jiang et al., 2020). In another study, the correlation between increase of TVB‐N of fish and Δ*E* changes of smart film based on starch/polyvinyl alcohol incorporated with roselle anthocyanins was indicated during storage at refrigerator temperature (Zhai et al., 2017).

**FIGURE 9:**
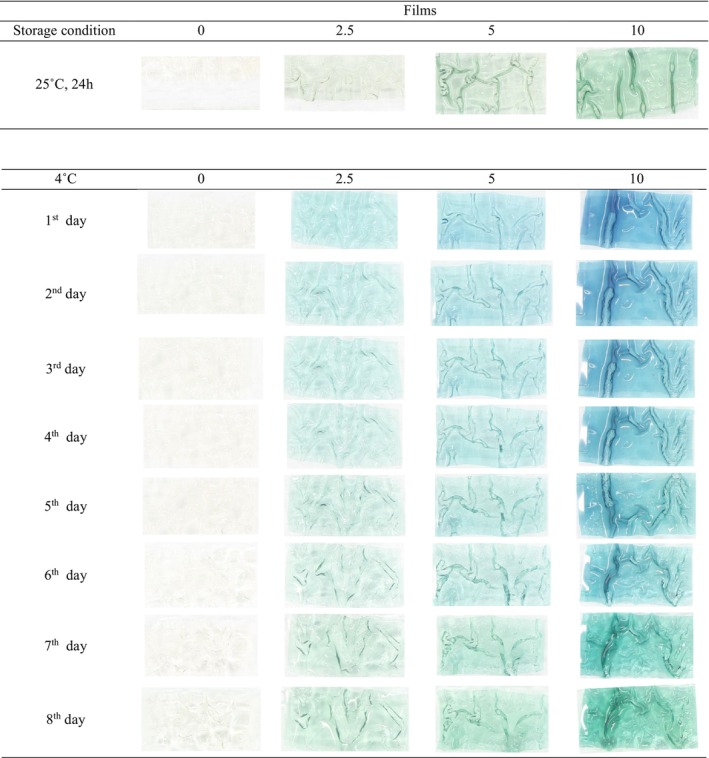
Color changes of composite BSG/chitosan films containing RCA (0, 2.5, 5, and 10 (v/v %)) during fish storage at 25°C and 4°C.

**FIGURE 10:**
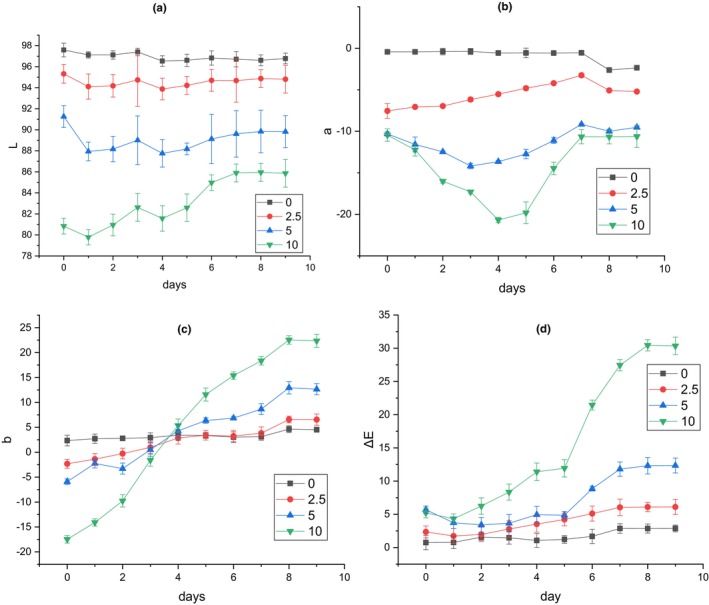
The variation of color indexes of smart films containing different concentrations of RCA during fish storage at 4°C: (a) L, (b) a, (c) b, and (d) Δ*E*.

The response of the smart films to the increase of volatile nitrogen compounds accumulated on the top of the container resulting from fish spoilage was observed in the form of color change. These visual color changes of the films on the packaging space are a useful indicator for the estimation of approximate microbial population and determination of fish sample spoilage (Kuswandi et al., 2012). In the present research, there was a correlation among microbial count, TVB‐N, and pH value of fish samples, and variation of color indexes of smart films during the storage period (Table 4). The pH value of fish samples increased to 10.8 at the end of cold storage (Figure 8). The changes in visual color and color indexes of the films during the fish storage period are demonstrated in Figures 9 and 10. At 25°C after 24 h, the color of the films at all three levels of RCA (2.5, 5, and 10% (v/v)) turned green while the intensity of color change was more in the film containing the highest RCA content. The control film (without RCA) did not show any color changes. The total color difference (Δ*E*) of films containing 2.5, 5, and 10% RCA after 24 h in the presence of spoiled fish significantly increased to 9.619, 12.182, and 27.051, respectively (*p* < .05) (Table 3). Also, the highest variation of *b* and Δ*E* indexes at the end of cold storage belonged to the smart film containing 10% RCA. Therefore, the amount of color change intensity of the smart films depended on the amount of anthocyanin level. Liu et al. (2021) also reported the green color change and increase of Δ*E* of RCA‐loaded polyvinyl alcohol/sodium carboxymethyl cellulose film after exposure to pork meat at 25°C for 24 h. Moreover, the color of smart films changed slightly toward green at 4°C with the storage time. Finally, the films clearly turned green on the 7th day (Figure 9) of fish cold storage. The Δ*E* also significantly increased with time in all three levels of the RCA 2.5%, 5%, and 10% to 6.086, 12.310, and 30.443, respectively (*p* < .05) (Figure 10). It should be noted that the film containing 10% extract exhibited a faster color change than other levels and the intensity of visible green color was higher (Figure 9).

**TABLE 3:** The color indexes of smart films containing different concentrations of red cabbage extract (RCA) after 24 h of fish storage at room temperature.

RCA (%)	L	a	b	Δ*E*
0	96.92 ± 0.027^a^	−0.438 ± 0.011^a^	2.99 ± 0.06^a^	1.41 ± 0.11^a^
2.5	93.65 ± 0.051^b^	−3.512 ± 0.051^b^	5.22 ± 0.44^b^	9.62 ± 0.63^b^
5	88.51 ± 0.421^c^	−8.712 ± 0.341^c^	6.02 ± 0.091^c^	12.18 ± 0.51^c^
10	82.492 ± 0.971^d^	−15.98 ± 0.114^d^	8.38 ± 0.487^d^	27.05 ± 0.23^d^

*Note*: Data are means ± SD (*n* = 3). Different letters in each column indicate a significant difference (*p* < .05) according to Duncan's means post hoc comparison.

**TABLE 4:** Pearson's correlation coefficients (*r*) among chemical and microbiological parameters of fish samples and total color difference (∆*E*) of smart films.

Variables	pH	TVC	TVBN	∆*E*
pH	–	0.989	0.932	0.948
TVC	0.989	–	0.959	0.959
TVBN	0.932	0.959	–	0.994
∆*E*	0.948	0.959	0.994	–

In earlier studies, other different polymer matrices were introduced as suitable carriers of natural anthocyanin dye. For instance, the starch/polyvinyl alcohol containing sour tea anthocyanins was successfully introduced as a homochromatic indicator of fish freshness. Wu et al. (2020) also observed the color change and Δ*E* enhancement of RCA‐loaded konjac glucomannan films from pink to green with an increase in pH. Jiang et al. (2020) reported that carboxymethylcellulose/starch film with sweet potato anthocyanins can be used as an indicator of fish freshness. They observed film color change from red to blue after spoilage of the fish sample and an increase of fish meat pH from 6.44 to 8.10. A bacterial cellulose nanofibers film enriched by black carrots anthocyanins as a pH‐sensitive film was applied to monitor fish freshness. The results indicated the potential of films to detect fish freshness after appearing in different colors including deep carmine color (fresh fish), charm pink color (the best time to consume fish), and jelly bean blue and khaki colors (rotten fish) (Moradi et al., 2019). Kuswandi et al. (2020) used RCA embedded in bacterial cellulose membrane as a pH‐sensitive tag to monitor the milk freshness. Fresh milk could be easily distinguished from spoiled milk after the tag color changes from blue‐gray to blue. Costa et al. (2020) reported that the color of the film prepared from jackfruit seed starch as a fish freshness indicator changed from pink to green after 24 h at room temperature. do Nascimento Alves et al. (2021) applied smart film based on green banana starch, gelatin, and alginate along with RCA to track the quality of sheep meat during storage. Along with the formation of volatile alkaline compounds in the samples during the storage period, the pH of meat was increased and the color parameters of the film were changed.

Generally, seafood spoilage is mainly due to microorganisms' activity and biochemical reactions. As the time of fish storage continues, various volatile nitrogen gases are produced that are slowly released from the fish pieces to the upper space of the storage container. Then, these compounds with an alkaline pH are absorbed by the smart film that is located in the container and can change the anthocyanin structure (Yan et al., 2021). The formation of chalcone, as one of the structural forms of anthocyanin during fish spoilage could be responsible for the green color of the smart films (Costa et al., 2020). In conclusion, these results indicated that the chitosan/BSG film containing red cabbage anthocyanin extract is very suitable for monitoring the freshness of fish through color change.

## 4 CONCLUSION

In the present study, the physicochemical, barrier, optical, and mechanical characteristics of fabricated composite films based on chitosan/BSG with various content of RCA (2.5, 5, and 10%) were investigated. Besides, the pH‐sensitive efficacy of smart films was investigated in vitro and in the presence of real food for food spoilage monitoring. The addition of red cabbage anthocyanin led to a decrease in moisture content and tensile strength and an increase in water solubility, WVP, opacity, Δ*E*, and elongation at the break of composite films. Also, the antioxidant potential of the film was enhanced after the incorporation of anthocyanin extract. The FTIR spectra confirmed the successful immobilization and interaction of red cabbage anthocyanin with the film structure. The SEM images confirmed improving compatibility between the film components in the presence of red cabbage anthocyanin. The smart films were able to change their color in different acidic, neutral, and alkaline environments, which suggested their application as an indicator for fish spoilage tracking at different temperatures. As a result, the highest intensity of obvious color change to green after fish spoilage was obtained by the smart film containing 10% anthocyanin extract. There was a correlation between TVC, TVB‐N, and Δ*E* changes of films during fish storage. Therefore, smart chitosan/BSG films can be introduced as nontoxic, biodegradable, and environmentally friendly packaging film matrices that can carry natural anthocyanin components and act as a pH‐sensitive indicator for monitoring food freshness during storage and determining the end point of food shelf life.

## AUTHOR CONTRIBUTIONS

**Maryam Nadi:** Data curation (lead); formal analysis (equal); methodology (equal); writing – original draft (lead). **Seyed Mohammad Ali Razavi:** Conceptualization (lead); funding acquisition (lead); project administration (lead); supervision (lead); validation (equal); writing – review and editing (equal). **Dina Shahrampour:** Conceptualization (supporting); formal analysis (supporting); methodology (supporting); validation (supporting); writing – review and editing (equal).

## FUNDING INFORMATION

This project was funded by the Deputy of Research, Ferdowsi University of Mashhad, Iran (Grant No. 56470), and the Iran National Science Foundation (INSF), Iran (Grant No., 96,015,540).

## CONFLICT OF INTEREST STATEMENT

The authors declare that they have no conflict of interest.

## Data Availability

The data that support the findings of this study are available from the corresponding author upon reasonable request.
